# Non-ST elevation acute coronary syndromes; clinical landscape, management strategy and in-hospital outcomes: an age perspective

**DOI:** 10.1186/s43044-021-00155-8

**Published:** 2021-03-31

**Authors:** Zainab Atiyah Dakhil, Hasan Ali Farhan

**Affiliations:** 1 Al-Kindy College of Medicine, University of Baghdad, Baghdad, Iraq; 2 Scientific Council of Cardiology, Iraqi Board for Medical Specializations, Baghdad, Iraq

**Keywords:** Quality of care, Age disparity, Healthcare equity, Audit, Elderly, GRACE score

## Abstract

**Background:**

As the elderly represent a substantial proportion of medical care beneficiaries, and there is limited data about age disparity in emerging countries, this study sought to investigate the impact of age on the management in patients with non-ST elevation acute coronary syndromes (NSTE-ACS).

**Results:**

Two hundred patients with NSTE-ACS enrolled prospectively, patients’ data, pharmacotherapy, management strategy as well timing to catheterization were documented. Patients grouped into ≥ 65 years versus < 65 years; 32.5% were ≥ 65-year-old. The older group presented as high GRACE risk (Global Registry of Acute Coronary Events) (67.7% versus 15.6%). Elderly patients were less likely to be referred for catheterization compared with younger counterparts (55.4% versus 76.3%, *p* = 0.003). Within low risk class patients, none of the elderly versus 9.33% of younger patients were catheterized within 2 h; in the same line, none of the elderly versus 16% of younger patients were catheterized within 24 h. Alternatively, at high risk class, 6.81% of the elderly and none of the younger patients were catheterized within 2 h. On the univariate analysis of variables to predict invasive strategy, presence of history of prior IHD, diabetes, absent in-hospital acute heart failure or atrial fibrillation/flutter, higher haemoglobin and lower creatinine levels predicted the use of invasive strategy, while on multivariate analysis, acute heart failure (95% CI − 0.38 to − 0.41, *p* = 0.01), lower haemoglobin (95% CI 0.002–0.07, *p* = 0.03), and atrial fibrillation/flutter (95% CI − 0.48 to − 0.02, *p* = 0.03) predicted conservative strategy. The elderly were more likely to have acute heart failure (32.3% versus 14.8%, *p* = 0.004), same as stroke (3.1% versus none, *p* = 0.04).

**Conclusions:**

Less-invasive strategy used in the elderly with NSTE-ACS compared with younger counterparts, yet age was not a predictor of catheterization underuse on multivariate analysis. It is crucial to bridge the age gap in the healthcare system in setting of ACS management by grasping the attention of decision makers and emphasizing on the adherence of healthcare providers to the guidelines to improve cardiovascular care and outcomes.

## Background

Healthcare disparities have recently raised the increasing amount of attention by researchers who suggested a wide range of inequity in the access to healthcare according to age, gender, socioeconomic status and ethnicity [1, 2]. Despite elderly patients having the highest absolute risk reduction from intervention in the context of acute coronary syndromes (ACS), there are other predictors that might impact decision-making and outcomes like frailty, cognitive impairment, comorbidities and bleeding risk [3]; however, age alone should not deprive patients from invasive management strategy especially that risk assessment tools on which the management plan is largely based like GRACE and TIMI scores consider age in risk estimation [4]. Taking in consideration the high penetration of age disparity in practice along with that elderly patients are becoming an increasingly substantial proportion of medical care beneficiaries [5], it became necessary to assess the presence as well as predictors for such disparity that can result frequently in treatment–risk paradox which will impact remarkably cardiovascular outcomes. Little is known about age disparity in the management of non-ST elevation acute coronary syndromes (NSTE-ACS) in emerging countries; in response, this study sought to investigate the impact of age on pharmacotherapy prescription, decision and timing of intervention in patients with NSTE-ACS.

## Methods

### Design

This is a prospective multicentre study.

### Setting and duration

The study was conducted in three teaching, percutaneous coronary intervention (PCI)-capable centres from January, 2018 to June, 2019; recruitment of cases was done sequentially during affiliation of the investigator to each cardiac centre.

### Patient selection

The study included patients who were diagnosed as NSTE-ACS (patients with acute chest pain with no persistent ST elevation) [6]. Exclusion criteria were persistent ST elevation in ECG, new or presumed new LBBB, active malignancy, end-stage renal disease or end-stage liver disease, recent history of upper GIT bleeding and patient refused invasive strategy.

### Data collection

A detailed history was taken from each patient including demographic features and comorbidities; results of in-hospital investigations were documented including ECG, echocardiography, haemoglobin, blood urea, serum creatinine and serum troponin. GRACE risk score was calculated with subsequent categorization of each patient into low, intermediate and high risk class according to the GRACE scores as the following: ≤ 108, 109–140 and > 140 respectively [4]. The patients were followed up during hospitalization with subsequent documentation of any of the following in-hospital complications: acute heart failure, recurrent or ongoing ischemic chest pain, cardiogenic shock, life-threatening arrhythmias, thromboembolic events (ischemic limb or pulmonary embolism), stroke and death.

### In-hospital management

Drugs prescribed during hospitalization were recorded for each patient as well as whether patient had catheterization during index hospitalization or not. For patients who were treated invasively, timing to catheterization was calculated and recorded.

Patients were grouped into two categories: those with age ˂ 65 years versus age ≥ 65 years. The baseline characteristics, comorbidities, prescribed drugs, management strategy (conservative versus invasive), timing to intervention and in-hospital outcomes were compared between the two categories.

### Primary and secondary outcomes

Primary outcome in this study was decision and timing of invasive strategy in patients according to their risk class. Secondary outcome was encountering any of the forementioned in-hospital complications.

### Coronary angiographic profile and revascularization recommendation

For patients who were catheterized during hospitalization, the results of coronary angiography were recorded; coronary lesions were classified into [7]

1. Patient with normal coronary arteries
2. Patients with non-critical coronary artery lesion(s): lesions causing < 70% of luminal narrowing
3. Patient with critical coronary artery lesion(s): lesions causing ≥ 70% luminal narrowing

If coronary revascularization (PCI versus coronary artery bypass graft (CABG)) was recommended by the treating team, then it was documented for each patient.

### Ethical approval

The study was performed in accordance with the declaration of Helsinki and approved by local ethical and scientific committee. Informed consent was obtained from all the participants.

### Statistical analysis

Collected data were coded and input into the computer using SPSS version 24. Numerical variables were expressed as mean ± standard deviation; categorical variables were expressed as percentages. Statistical analysis of numerical variables was done by *t* test, while that of categorical variables was done by chi-squared test to compare frequency ratios between categories. Multiple regression analysis model was structured to study the independent predictors of using invasive strategy. *p* value < 0.05 is considered statistically significant.

## Results

### Cohort baseline characteristics

A total of 200 patients were enrolled, of whom 32.5% were ≥ 65 years old; mean age of the older group was 71.82 ± 6.03 years compared with 52.46 ± 8.09 years in the younger group (*p* < 0.0001); 44.6% of the patients were females in the older group versus 22.2% in younger patients (*p* = 0.001). Hypertension (HT) and diabetes (DM) were more evident in the older group (84.6 and 61.5% respectively), while younger patients were more smokers (42.2% versus 18.5%). Elderly patients were more likely to be presented with dyspnoea (38.5% versus 25.2%) (*p* = 0.054), and they were presented as high GRACE risk class (67.7% versus 15.6%), while younger counterparts were presented more as low GRACE risk class (55.6% versus 13.8%) *p* < 0.0001. Elderly patients had higher blood urea (47.2 ± 20.6 versus 35.6 ± 15.05, *p* < 0.0001) and higher serum creatinine (1.04 ± 0.3 versus 0.9 ± 0.3, *p* = 0.01), while they had lower haemoglobin than their younger counterparts (12.7 ± 1.9 versus 13.7 ± 2.06, *p* = 0.006); these results can be seen in Table 1.

**Table 1:** Baseline characteristics in patients with NSTE-ACS according to age category

Baseline characteristics	<65 Years	≥ 65 Years	***p*** value
	*N* (%)		
*N* (%)	135 (67.5%)	65 (32.5%)	-
Age (mean ± SD) years	52.46 ± 8.09	71.82 ± 6.03	<0.0001
Female gender	30 (22.2%)	29 (44.6%)	0.001
Hypertension	84 (62.2%)	55 (84.6%)	0.001
Diabetes	58 (43%)	40 (61.5%)	0.014
IHD	57 (42.2%)	39 (60%)	0.018
Family history	44 (32.6%)	21 (32.3%)	0.9
Smoking	57 (42.2%)	12 (18.5%)	0.001
Hyperlipidaemia	26 (19.3%)	19 (29.2%)	0.1
Stroke	3 (2.2%)	3 (4.6%)	0.3
Atrial fibrillation (new or old)	9 (6.7%)	8 (12.3%)	0.1
Prior catheterization	9 (6.7%)	8 (12.3%)	0.18
Prior PCI	14 (21.5%)	21 (15.6%)	0.29
Prior CABG	1 (0.7%)	4 (6.2%)	0.02
Dyspnoea	34 (25.2%)	25 (38.5%)	0.054
Positive troponin	57 (42.2%)	36 (55.4%)	0.08
Pulse rate (mean ± SD) BPM	84.3 ± 21.5	85.8 ± 22.3	0.6
SBP (mean ± SD) mmHg	135.3 ± 24.3	139 ± 27.4	0.3
DBP (mean ± SD) mmHg	82.2 ± 13.2	79.03 ± 11.7	0.09
Ejection fraction	54.8 ± 11.6	51.6 ± 11.9	0.1
Blood sugar	182.6 ± 110.1	197.08 ± 109.9	0.4
Urea (mean ± SD) mg/dL	35.6 ± 15.05	47.2 ± 20.6	<0.0001
Creatinine (mean ± SD) Mg/dL	0.9 ± 0.3	1.04 ± 0.3	0.01
Estimated GFR^a^ (mean ± SD) ml/min/1.73 m^2^	90.75 ± 20.11	67.4 ± 20.13	<0.0001
Haemoglobin (mean ± SD) gm/L	13.7 ± 2.06	12.7 ± 1.9	0.006
WBC (mean ± SD) × 10^3^/mcL	9.369 ± 3.027	9.362 ± 2.859	0.9
Platelets count (mean ± SD)/mcL	235020 ± 77.075	236250 ± 76.375	0.9
Hospitalization duration (mean ± SD) days	4.9 ± 2.7	4.8 ± 2.8	0.7
**GRACE risk class**			
Low	75 (55.6%)	9 (13.8%)	<0.0001
Intermediate	39 (28.9%)	12 (18.5%)	
High	21 (15.6%)	44 (67.7%)	

^a^GFR estimated by CKD-EPI creatinine equation

### In-hospital management

#### Pharmacotherapy

Both groups were treated at the same rate with aspirin 98.5%, while P2Y12 inhibitors were used in all elderly 100% and most younger patients 99.3%; heparin was used less in the elderly, same as B-blockers, ACEI/ARBs and statins; however, there is no statistical difference between two groups regarding pharmacotherapy during hospitalization apart from mineralocorticoid receptor antagonist (MRA) which was used in the elderly in 18.5% versus 6.7% in younger counterparts (*p* = 0.01); these results are shown in Table 2.

**Table 2:** Pharmacotherapy profile in patients with NSTE-ACS according to age category

Medication	<65 Years	≥ 65 Years	***p*** value
	*N* (%)		
Aspirin^a^	133 (98.5%)	68 (98.5%)	0.9
P2Y12 inhibitor	134 (99.3%)	65 (100%)	0.4
Heparin	97 (71.9%)	46 (70.8%)	0.8
B-blockers	105 (77.8%)	49 (75.4%)	0.7
Nitrate	80 (59.3%)	42 (64.6%)	0.4
Calcium channel blockers	11 (8.1%)	10 (15.4%)	0.1
ACEI/ARBs	57 (42.2%)	27 (41.5%)	0.9
Statin	123 (91.1%)	53 (81.5%)	0.051
Mineralocorticoid receptor antagonist	9 (6.7%)	12 (18.5%)	0.01
Loop diuretics	21 (15.6%)	16 (24.6%)	0.1

^a^Including a patient with dipyridamole use instead of aspirin because of history of aspirin allergy

#### Coronary catheterization and timing to intervention

**Fig. 1:**
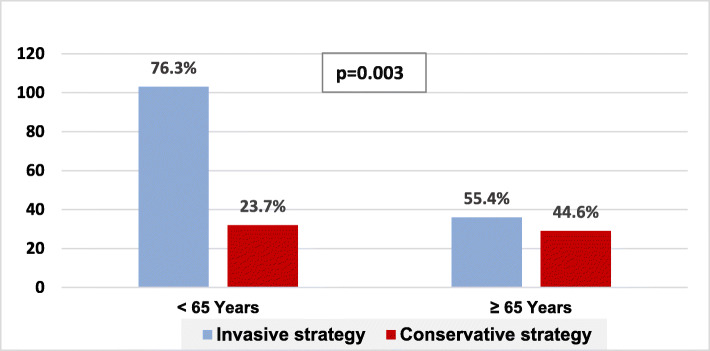
Management strategy in NSTE-ACS according to age category

**Fig. 2:**
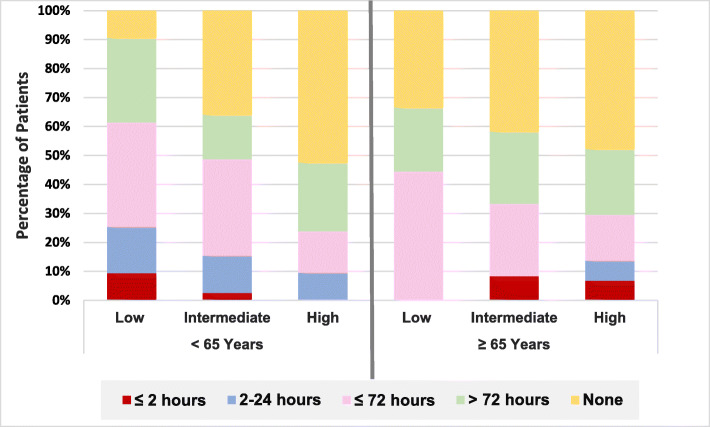
Timing to catheterization in NSTE-ACS according to age and GRACE risk class. Comparing timing to catheterization between the two age categories according to risk class *p* value = 0.4 (low-risk class), 0.3 (intermediate-risk class) and 0.6 (high risk class)

Elderly patients were less likely to be referred for catheterization compared with the younger counterparts (55.4% versus 76.3%, *p* = 0.003); as seen in Fig. 1, within each age category, when considering GRACE risk class, the highest non-catheterization rate was seen in high risk class in both elderly and younger patients (47.72% and 52.38%) respectively. Regarding timing to intervention in view of each risk class, within the low-risk-class patients, none of the elderly versus 9.33% of the younger patients were catheterized within 2 h; in the same line, none of the elderly versus 16% of the younger patients were catheterized within 24 h. Alternatively, at high risk class, 6.81% of the elderly and none of the younger patients were catheterized within 2 h, while 22.72% of the older patients and 32.8% of the younger patients were catheterized within more than 72 h, as illustrated in Fig. 2. On univariate analysis of variables to predict invasive strategy, presence of history of prior IHD, diabetes, absent in-hospital acute heart failure or atrial fibrillation/flutter, higher haemoglobin and lower creatinine levels predicted the use of invasive strategy, while on multivariate analysis, acute heart failure (95% CI − 0.38 to − 0.41, *p* = 0.01), lower haemoglobin (95% CI 0.002–0.07, *p* = 0.03) and atrial fibrillation/flutter (95% CI − 0.48 to − 0.02, *p* = 0.03) predicted conservative strategy. The elderly were more likely to have acute heart failure (32.3% versus 14.8%, *p* = 0.004), same as stroke (3.1% versus none, *p* = 0.04). See Tables 3 and 4.

**Table 3:** Univariate analysis of predictors of invasive strategy in NSTE-ACS

Variable	Conservative strategy	Invasive strategy	***p*** value
Age ≥ 65 years	29 (44.6 %)	36 (55.4%)	0.003
Female gender	22 (37.3%)	37 (62.7%)	0.17
IHD	38 (39.6%)	58 (60.4%)	0.007
Diabetes	38 (38.8%)	60 (61.2%)	0.013
Smoking	16 (23.2%)	53 (76.8%)	0.103
Positive troponin	30 (32.3%)	63 (67.7%)	0.61
Acute heart failure	24 (58.5%)	17 (41.5%)	<0.0001
Cardiogenic shock	4 (66.7%)	2 (33.3%)	0.051
Ongoing chest pain	18 (36.7%)	31 (63.3%)	0.27
AF/A FL	11 (64.7%)	6 (35.3%)	0.001
Arrhythmias^a^	3 (30%)	7 (70%)	0.97
Haemoglobin	12.79 ± 2.37	13.64 ± 1.87	0.014
Creatinine	1.03 ± 0.43	0.92 ± 0.24	0.03

^a^Life-threatening arrhythmias

**Table 4:** Multi-regression analysis of predictors of invasive strategy in NSTE-ACS^*^

Predictors	Standard error	Standardized coefficient (beta)	95% confidence interval		***p*** value
			Lower	Upper	
Age ≥ 65 years	0.07	− 0.101	− 0.25	0.05	0.2
Female gender	0.08	0.05	− 0.11	0.22	0.52
IHD	0.06	− 1	− 0.22	0.04	0.19
Diabetes	0.07	− 0.07	− 0.20	0.07	0.33
Smoking	0.07	0.05	− 0.09	0.2	0.48
Positive troponin	0.06	− 0.001	− 0.13	0.13	0.99
Acute heart failure	0.09	− 0.2	− 0.38	− 0.41	0.01
Cardiogenic shock	0.2	− 0.12	− 0.73	0.06	0.09
Ongoing chest pain	0.08	0.02	− 0.14	0.18	0.78
AF/A FL	0.11	− 0.16	− 0.48	− 0.02	0.03
Arrhythmias^a^	0.15	− 0.003	− 0.31	0.306	0.97
Haemoglobin	0.01	0.17	0.002	0.07	0.03
Creatinine	0.11	− 0.05	− 0.308	0.14	0.46

^a^Life-threatening arrhythmias

^*^p value of the multiple regression model is < 0.0001

#### Coronary angiographic findings and recommended revascularization mode

**Fig. 3:**
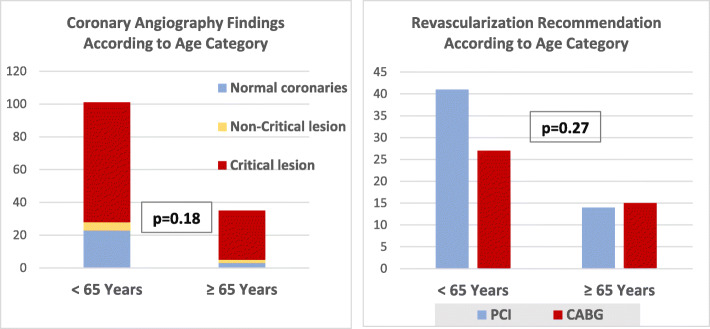
Coronary angiography findings and revascularization recommendation in NSTE-ACS according to age category. Among total patients referred for catheterization (*n* = 139), catheterization records were missed in 3 patients (1 in age < 65 years and 2 in older counterparts)

Among patients who were referred for catheterization, critical coronary lesions were recorded more in the elderly group (85.71% versus 72.27%), while normal coronary angiography was evident more in the younger group (22.77% versus 8.57%); however, there were no statistically significant differences between the two groups regarding coronary findings nor revascularization recommendation (PCI versus CABG); these findings are shown in Fig. 3.

### In-hospital complications and outcomes

**Fig. 4:**
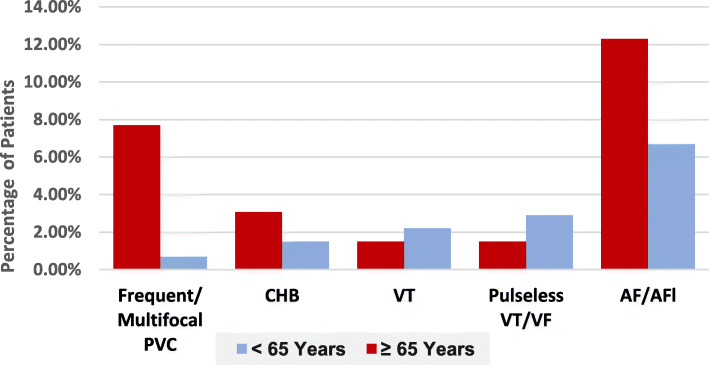
Distribution of arrhythmias in NSTE-ACS according to age category. Patients who had more than one type of arrhythmia recorded within each type separately

**Fig. 5:**
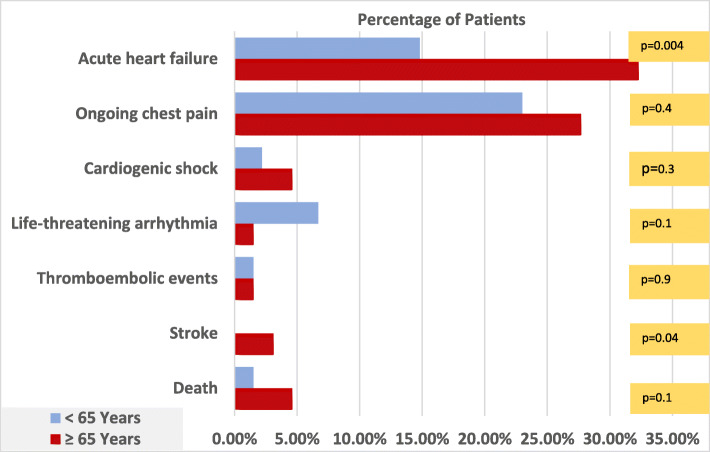
In-hospital complications in NSTE-ACS patients a according to age category

Older patients were more likely to have acute heart failure (AHF) than younger counterparts (32.3% versus 14.8%, *p* = 0.004), same as stroke (3.1% in elderly and none in younger counterparts, *p* = 0.04), while younger patients tend to develop life-threatening arrhythmias (6.7% versus 1.5%, p=0.1) more. Atrial fibrillation (AF) and premature ventricular contractions (PVC) were most common arrhythmias in older patients (12.3 and 7.7% respectively). In-hospital death occurred more in older patients (4.6% versus 1.5%, *p* = 0.1); these findings are revealed in Figs. 4 and 5.

### Duration of hospitalization

**Fig. 6:**
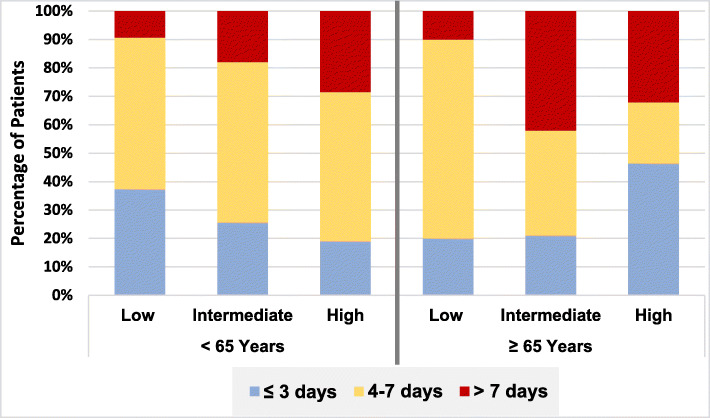
Hospitalization duration in NSTE-ACS according to age and GRACE risk class. Comparing hospitalization duration between the two age categories according to risk class *p* value = 0.3 (low-risk class), 0.6 (intermediate-risk class) and 0.51 (high-risk class)

At the low risk class, elderly patients and younger patients were more likely to be hospitalized for 4–7 days in 70 and 53.33% respectively; at the high risk class, the older group was hospitalized more for ≤ 3 days (46.42%), while younger counterparts were hospitalized more for 4–7 days (52.38%), as shown in Fig. 6.

## Discussion

Despite the limited data about adherence of cardiologists to international guidelines in Iraq, it is only recently that healthcare disparities’ impact on decision-making have been addressed [8, 9]; this called us for conducting further studies that cast a light on healthcare disparities including age disparity and its predictors [10] to inform stakeholders, hoping to respond by establishing quality improvement projects to bridge disparity gaps in practice. In response, the current study came to bring into view the underutilization of invasive strategy in elderly patients with NSTE-ACS, yet, it contradicted prior researches that suggested underuse of invasive strategy in elderly patients with high GRACE risk class despite most prognostic benefit [11] as current study revealed more conservative treatment in higher-risk patients in all patients in general and the younger age group in particular; the study contributes also by giving insight to timing of intervention and duration of hospitalization within the age categories according to GRACE risk class, an issue about which we have limited data.

Elderly patients are under-represented in clinical trials resulting in uncertainty of clinicians about impact and benefit of many therapeutic options in this population [12, 13] leading to under-prescription of guideline-directed medical therapy (GDMT) in the setting of ACS; among factors that contribute to this under-prescription is the age-related pathophysiological changes resulting in altering pharmacokinetic and pharmacodynamic responses to drugs, the low weight that commonly occurs in elderly, increased bleeding events with use of antithrombotic and antiplatelet therapies, commonly associated anaemia and other comorbidities, and polypharmacy with subsequent drugs interaction [14–17]. However, current study showed more universal use of aspirin and P2Y12 inhibitors in both age groups compared with prior studies [12, 13, 18]. Despite guideline recommendations [19–21] and prognostic benefit, there is a trend to use statin more in the younger group, consistent with earlier studies [12, 18]. While aging did not impact their prescription, B-blockers and ACEI/ARBs were used at a lower rate in the current study compared with earlier researches [12, 13]; B-blockers and ACEI/ARBs were expected to be used more significantly compared with the younger group in our study as the recruited elderly patients had more indications to use ACEI/ARBs in terms of being more diabetic, hypertensive with higher systolic blood pressure, have lower ejection fraction and remarkably developed heart failure during hospitalization more than their younger counterparts, yet the older study group had higher renal indices which might explain the relative under-prescription of ACEI/ARBs fearing from developing hyperkalaemia, in addition to potential complications of postural hypotension and increased risk of falls; however, such adverse effects can be overcome by proper monitoring of renal function and gradual up titration of doses [15, 22]. In the same line, fearing from adverse effects of B-blockers like fatigue, dizziness, or physicians’ perception of less efficacy in the elderly due to decrease B-receptors [13, 23], all can lead to under use of B-blockers in the older group in spite of their better prognostic effect in the elderly compared with younger counterparts in terms of reducing death and recurrent myocardial infarction [19–21]. Impaired left ventricular function and higher incidence of acute heart failure justify the higher rate of using loop diuretic and mineralocorticoid receptor antagonist in the older group. Older patients were less treated invasively; these results were supported by prior studies including GRACE and ROSAI-2 registries [13, 24–27]. Many factors contribute to less referral for catheterization in older patients with NSTE-ACS like associated comorbidities, especially anaemia and CKD, higher rate of bleeding complications and the more presentation with haemodynamic instability in this population [13]. Furthermore, there are contradictory results with respect to prognostic benefit of routine invasive strategy in the elderly with NSTE-ACS, MOSCA and Italian Elderly ACS trials [28, 29], which are two randomized controlled trials that showed no difference between invasive and conservative strategies in the elderly with NSTE-ACS in terms of recurrent MI, cardiovascular rehospitalization and mortality. However, After-Eighty trial, TACTICS-TIMI18 trial [30, 31] and other meta-analyses [32, 33] revealed decrease in recurrent MI, need for revascularization and death in elderly patients who were treated with early invasive strategy. Other meta-analysis showed less need for revascularization in the invasive strategy with no significant difference in all-cause mortality, cardiovascular mortality, stroke and MI [34]. GRACE registry also revealed mortality benefit of invasive strategy at 6 months follow-up but with no difference in MI rate [26]. Similarly, a real-life cohort study revealed lower in-hospital complications (AF, HF, stroke and death) same as lower long-term mortality with invasive strategy, while having no change regarding in-hospital cardiogenic shock, AV block and VT/VF, yet invasive strategy was associated remarkably with higher bleeding complications [35]. What can be abstracted from earlier studies is that it is not only chronological age that should determine the decision of intervention but also other factors like frailty, comorbidity, functional status and life expectancy.

Current study compared timing to catheterization between both age categories according to their GRACE risk class, despite that patients at low and intermediate GRACE risk classes were more to be catheterized in the younger age group, while at high GRACE risk class, the elderly were catheterized more and at earlier timing than their younger counterparts; however, the rate of use of early invasive strategy in high GRACE risk class in total is much lower than reported in literature [36] despite the better prognostic benefit with early invasive strategy (within 24 h) in high-risk NSTE-ACS in terms of lower death, MI, and stroke as suggested by TIMACS trial [37] and ACUITY trial [38].

Current study showed that there are higher rates of critical coronary lesions in the elderly and higher rates of normal coronary angiography in the younger counterparts, while older patients were referred for CABG more than the younger group indicating higher-risk coronary anatomy in the elderly (left main stem or three-vessel disease). Prior researchers also showed higher normal coronary angiography in younger ACS patients as well as higher-risk coronary anatomy with increasing age, yet there is lower referral for PCI or CABG in older patients [39].

The study disclosed a higher rate of in-hospital complications in elderly patients in terms of acute heart failure and stroke. Interestingly, life-threatening arrhythmias were reported more in the younger group, while AF and multifocal PVC occurred more in the older counterparts; these results were supported by prior researchers [40] as higher rate of arrhythmias, HF, cardiogenic shock and death were recorded in older patients, while other researchers reported higher HF, recurrent MI and life-threatening arrhythmias in younger patients in the time that AF, cardiogenic shock and stroke recorded more in the older group; however, these changes did not reach statistical significance [41]. The current study showed no significant difference in average duration of hospitalization according to age categories nor duration of hospitalization between the two age groups according to the GRACE risk class; results are consistent with prior researchers [42] and contradicted others [43]. The ESC guideline recommended discharging patients with NSTE-ACS within 48–72 h for patients at low risk class [44], while the current study revealed that the highest rate of patients among all GRACE risk classes in the younger group and low GRACE risk class in the older age were hospitalized for 4–7 days, while high-GRACE risk patients of the older age group were more likely to be hospitalized for ≤ 3 days; this short period in high-GRACE risk elderly might be due to more use of invasive strategy in them compared to high-GRACE risk younger counterparts. Despite prior studies, observed variables like lower comorbidities, higher use of ACEI/ARBs, lower use of loop diuretics and MRA, and higher left ventricular EF in those with shorter duration of hospitalization [42, 43], such factors were significantly different between the two age groups in our study, yet it seems that they did not signify duration of hospitalization, hence no significant difference between the two age categories. Earlier discharge of NSTE-ACS patients particularly those of low- and intermediate-GRACE risk classes is crucial considering issues like cost-effectiveness, patients’ satisfaction, prevention of bed blocking especially in PCI-capable centres, where work flow can be affected remarkably by limited bed capacity which can impact other patients’ cardiovascular care and outcomes.

This study provides insight to age differences in the current practice of managing NSTE-ACS which could be due to multiple comorbidities, higher GRACE risk class at presentation and anticipating complications; however, these differences can be overcome by proper risk stratification, adherence to international guidelines in addition to trying to minimize complications like prescribing PPI and using radial approach to decrease bleeding risk as well as GFR estimation and proper assessment of contrast nephropathy risk to minimize it by rehydration and limiting contrast amount. Thus, chronological age alone will not deprive the patient from being managed as indicated because precise risk–benefit assessment will be implemented.

Study limitations are as follows: a larger study population is needed to further validate the statistical results; small sample size was mainly due to absent electronic data base in our healthcare facilities and limited research collaborators. Prognostic impact of using invasive strategy and timing of intervention to achieve most benefit was not assessed in the current study despite being controversial in literature.

## Conclusions

Despite elderly patients with NSTE-ACS presented at higher GRACE risk class and despite the robust guidelines’ recommendations, there is an age gap in managing this population in the form of underutilization of invasive strategy depriving them from its prognostic benefit; however, age was not a predictor of invasive strategy underuse on multivariate analysis which suggested that other factors like developing acute heart failure or presence of AF or having lower haemoglobin levels may play a role in determining management strategy. Considering the substantial increase in geriatric population in different societies, it is crucial to bridge the age gap in healthcare system in the setting of ACS management by grasping the attention of decision makers and emphasizing on the adherence of healthcare providers to the guidelines to improve cardiovascular care and outcomes.

## Data Availability

The data sets used and/or analysed during the study are available from the corresponding author on reasonable request.

## Abbreviations

NSTE-ACS: Non-ST elevation acute coronary syndromes
GRACE: Global Registry of Acute Coronary Events
ACS: Acute coronary syndromes
AHF: Acute heart failure
AF: Atrial fibrillation
CABG: Coronary artery bypass graft
DM: Diabetes mellitus
GDMT: Guideline-directed medical therapy
HT: Hypertension
MRA: Mineralocorticoid receptor antagonist
PCI: Percutaneous coronary intervention
PVC: Premature ventricular contraction
AFL: Atrial flutter

## References

[1] Yamada T, Chen CC, Murata C, Hirai H, Ojima T, Kondo K, Joseph RH (2015). Access disparity and health inequality of the elderly: unmet needs and delayed healthcare. Int J Environ Res Public Health.

[2] Riley WJ (2012). Health disparities: gaps in access, quality and affordability of medical care. Trans Am Clin Climatol Assoc.

[3] Kumar S, McDaniel M, Samady H, Forouzandeh F (2020) Contemporary Revascularization dilemmas in older adults. J Am Heart Assoc 9(3):e014477. 10.1161/JAHA.119.014477 10.1161/JAHA.119.014477 PMC7033869 31973608

[4] de Araújo GP, Ferreira J, Aguiar C, Seabra-Gomes R (2005) TIMI, PURSUIT, and GRACE risk scores: sustained prognostic value and interaction with revascularization in NSTE-ACS. Eur Heart J 26(9):865–872. 10.1093/eurheartj/ehi187 10.1093/eurheartj/ehi187 15764619

[5] Hazra NC, Rudisill C, Gulliford MC (2018). Determinants of health care costs in the senior elderly: age, comorbidity, impairment, or proximity to death?. Eur J Health Econ.

[6] Roffi M, Patrono C, Collet JP, Mueller C, Valgimigli M et al (2016) 2015 ESC guidelines for the management of acute coronary syndromes in patients presenting without persistent ST segment elevation. Task Force for the Management of Acute Coronary Syndromes in Patients Presenting without Persistent ST-Segment Elevation of the European Society of Cardiology (ESC). Eur Heart J 37:267–315. 10.1093/eurheartj/ehv320 10.1093/eurheartj/ehv320 26320110

[7] Crenshaw JH, Sullivan JM, Ramanathan KB, Mirvis DM, El-Zeky F, Vander ZR (1995). The effect of noncritical coronary artery disease on long-term survival. Am J Med Sci.

[8] Dakhil Z, Farhan HA (2020) Treatment risk paradox in non ST elevation acute coronary syndromes: Is it a matter of gender gap? J Am Coll Cardiol 75(11 Supplement 1):31. 10.1016/S0735-1097(20)30658-6

[9] Dakhil Z, Farhan HA (2020) Off-hours admission of patients with non ST elevation acute coronary syndromes: Does it impact the management strategy? Catheter Cardiovasc Interv 95(52):S1–S229. 10.1002/ccd.28864

[10] Dakhil Z, Farhan HA (2020) Underuse of invasive strategy in elderly patients with non-ST elevation acute coronary syndromes: Is it real? Eur Heart J Suppl 22(Supplement_K):K12. 10.1093/eurheartj/suaa120

[11] Bagnall AJ, Goodman SG, Fox KA, Yan RT, Gore JM, Cheema AN, Huynh T, Chauret D, Fitchett DH, Langer A, Yan AT, Canadian Acute Coronary Syndrome Registry I and II Investigators, Canadian Global Registry of Acute Coronary Events (GRACE/GRACE2) Investigators (2009). Influence of age on use of cardiac catheterization and associated outcomes in patients with non-ST-elevation acute coronary syndromes. Am J Cardiol.

[12] Zaman MJ, Stirling S, Shepstone L, Ryding A, Flather M, Bachmann M, Myint PK (2014). The association between older age and receipt of care and outcomes in patients with acute coronary syndromes: a cohort study of the Myocardial Ischaemia National Audit Project (MINAP). Eur Heart J.

[13] Orenes-Piñero E, Ruiz-Nodar JM, Esteve-Pastor MA, Quintana-Giner M, Rivera-Caravaca JM, Veliz A, Valdés M, Macías M, Pernias-Escrig V, Vicente-Ibarra N, Carrillo L, Sandín-Rollán M, Candela E, Lozano T, Marín F (2017). Therapeutic management and one-year outcomes in elderly patients with acute coronary syndrome. Oncotarget..

[14] Ayan M, Pothineni NV, Siraj A, Mehta JL (2016) Cardiac drug therapy-considerations in the elderly. J Geriatr Cardiol 13:992–997. 10.11909/j.issn.1671-5411.2016.12.008. 10.11909/j.issn.1671-5411.2016.12.008 PMC5351831 28321243

[15] Martinez BK, White CM (2019). Pharmacologic considerations in the management of acute coronary syndrome in elderly patients. Expert Opin Pharmacother.

[16] Alexander KP, Newby LK, Cannon CP, Armstrong PW, Gibler WB, Rich MW, van de Werf F, White HD, Weaver WD, Naylor MD, Gore JM, Krumholz HM, Ohman EM, American Heart Association Council on Clinical Cardiology, Society of Geriatric Cardiology (2007). Acute coronary care in the elderly, part I: non-STsegment-elevation acute coronary syndromes: a scientific statement for healthcare professionals from the American Heart Association Council on Clinical Cardiology: in collaboration with the Society of Geriatric Cardiology. Circulation..

[17] Alexander KP, Newby LK, Armstrong PW, Cannon CP, Gibler WB, Rich MW, van de Werf F, White HD, Weaver WD, Naylor MD, Gore JM, Krumholz HM, Ohman EM, American Heart Association Council on Clinical Cardiology, Society of Geriatric Cardiology (2007). Acute coronary care in the elderly, part II: ST-segment-elevation myocardial infarction: a scientific statement for healthcare professionals from the American Heart Association Council on Clinical Cardiology: in collaboration with the Society of Geriatric Cardiology. Circulation..

[18] Capodanno D, Angiolillo DJ (2010). Antithrombotic therapy in the elderly. J Am Coll Cardiol.

[19] Collet JP, Thiele H, Barbato E, Barthélémy O, Bauersachs J, Bhatt DL, et al, ESC Scientific Document Group (2020) 2020 ESC Guidelines for the management of acute coronary syndromes in patients presenting without persistent ST-segment elevation. Eur Heart J. 10.1093/eurheartj/ehaa575. (Epub ahead of print. PMID: 32860058) 10.1093/eurheartj/ehaa575 32860058

[20] O’Gara PT, Kushner FG, Ascheim DD, Casey DE, Chung MK (2013). 2013 ACCF/AHA guideline for the management of ST-elevation myocardial infarction: a report of the American College of Cardiology Foundation/American Heart Association Task Force on Practice Guidelines. J Am Coll Cardiol.

[21] Amsterdam EA, Wenger NK, Brindis RG, Casey DE, Ganiats TG (2014). 2014 AHA/ACC guideline for the management of patients with non-ST-elevation acute coronary syndromes: executive summary: a report of the American College of Cardiology/American Heart Association Task Force on Practice Guidelines. Circulation..

[22] Carroll KA, Early NK, Tsu LV (2015). Managing acute coronary syndromes in the elderly. Consult Pharm.

[23] Del Sindaco D, Tinti MD, Monzo L, Pulignano G (2010). Clinical and economic aspects of the use of nebivolol in the treatment of elderly patients with heart failure. Clin Interv Aging.

[24] Buber J, Goldenberg I, Kimron L, Guetta V (2013). One-year outcome following coronary angiography in elderly patients with non-ST elevation myocardial infarction: real-world data from the Acute Coronary Syndromes Israeli Survey (ACSIS). Coron Artery Dis.

[25] Di Bari M, Balzi D, Fracchia S, Barchielli A, Orso F (2014). Decreased usage and increased effectiveness of percutaneous coronary intervention in complex older patients with acute coronary syndromes. Heart..

[26] Devlin G, Gore JM, Elliott J, Wijesinghe N, Eagle KA, Avezum A, Huang W, Brieger D, GRACE Investigators (2008). Management and 6-month outcomes in elderly and very elderly patients with high-risk non-ST-elevation acute coronary syndromes: The Global Registry of Acute Coronary Events. Eur Heart J.

[27] De Servi S, Cavallini C, Dellavalle A, Santoro GM, Bonizzoni E (2004). ROSAI-2 Investigators. Non-ST–elevation acute coronary syndrome in the elderly: treatment strategies and 30-day outcome. Am Heart J.

[28] Sanchis J, Núñez E, Barrabés JA, Marín F, Consuegra-Sánchez L, Ventura S, Valero E, Roqué M, Bayés-Genís A, del Blanco BG, Dégano I, Núñez J (2016). Randomized comparison between the invasive and conservative strategies in comorbid elderly patients with non-ST elevation myocardial infarction. Eur J Intern Med.

[29] Savonitto S, Cavallini C, Petronio AS, Murena E, Antonicelli R, Sacco A, Steffenino G, Bonechi F, Mossuti E, Manari A, Tolaro S, Toso A, Daniotti A, Piscione F, Morici N, Cesana BM, Jori MC, de Servi S, Italian Elderly ACS Trial Investigators (2012). Early aggressive versus initially conservative treatment in elderly patients with non-ST-segment elevation acute coronary syndrome: a randomized controlled trial. JACC Cardiovasc Interv.

[30] Tegn N, Abdelnoor M, Aaberge L, Endresen K, Skårdal R (2016). Invasive versus conservative strategy in patients aged 80 years or older with non-ST-elevation myocardial infarction or unstable angina pectoris (After Eighty study): an open-label randomised controlled trial. Lancet..

[31] Bach RG, Cannon CP, Weintraub WS, DiBattiste PM, Demopoulos LA (2004). The effect of routine, early invasive management on outcome for elderly patients with non–ST-segment elevation acute coronary syndromes. Ann Intern Med.

[32] Angeli F, Verdecchia P, Savonitto S, Morici N, De Servi S, Cavallini C (2014). Early invasive versus selectively invasive strategy in patients with non-ST-segment elevation acute coronary syndrome: impact of age. Catheter Cardiovasc Interv.

[33] Garg A, Garg L, Agarwal M, Rout A, Raheja H, Agrawal S, Rao SV, Cohen M (2018). Routine invasive versus selective invasive strategy in elderly patients older than 75 years with non-ST-segment elevation acute coronary syndrome: a systematic review and meta-analysis. Mayo Clin Proc.

[34] Reaño JD, Shiu LA, Miralles KV, Dimalala MG, Pestaño NS (2020). A systematic review and meta-analysis on the effectiveness of an invasive strategy compared to a conservative approach in patients > 65 years old with non-ST elevation acute coronary syndrome. PLoS One.

[35] Kvakkestad KM, Gran JM, Eritsland J, Hansen CH, Fossum E, Andersen GØ, Halvorsen S (2019). Long-term survival after invasive or conservative strategy in elderly patients with non-ST-elevation myocardial jnfarction: a prospective cohort study. Cardiology..

[36] Badings EA, Hermanides RS, Van Der Sluis A, Dambrink JH, Gosselink AT (2019). Use, timing and outcome of coronary angiography in patients with high-risk non-ST-segment elevation acute coronary syndrome in daily clinical practice: insights from a ‘real world’ prospective registry. Neth Hear J.

[37] Afzal R, Chrolavicius S, Jolly SS, Widimsky P, Avezum A et al (2009) Early versus delayed invasive intervention in acute coronary syndromes. N Engl J Med 360:2165–2175. 10.1056/nejmoa0807986 10.1056/NEJMoa0807986 19458363

[38] Costantini C, Stuckey T, Tcheng JE, Mehran R, Lansky AJ, Grines CL, Stone GW (2010) Impact of delay to angioplasty in patients with acute coronary syndromes undergoing invasive management: analysis from the ACUITY (Acute Catheterization and Urgent Intervention Triage strategY) trial. J Am Coll Cardiol 55:1416–1424. 10.1016/j.jacc.2009.11.063 10.1016/j.jacc.2009.11.063 20359590

[39] Rosengren A, Wallentin L, Simoons M, Gitt AK, Behar S, Battler A, Hasdai D (2006). Age, clinical presentation, and outcome of acute coronary syndromes in the Euroheart acute coronary syndrome survey. Eur Heart J.

[40] Mirghani HO (2016) Age related differences in acute coronary syndrome presentation and in hospital outcomes: a cross-sectional comparative study. Pan Afr Med J 24. 10.11604/pamj.2016.24.337.8711 10.11604/pamj.2016.24.337.8711 PMC5267853 28154692

[41] Su PH, Chen PY, Lee SY, How CK, Chien DK, Chang WH (2019) Comparison of clinical presentations and outcomes between adult and elderly acute myocardial infarction patients in emergency department. Heal Technol 3:7. 10.21037/ht.2019.08.01

[42] Węgiel M, Dziewierz A, Wojtasik-Bakalarz J, Sorysz D, Surdacki A, Bartuś S, Dudek D, Rakowski T (2018). Hospitalization length after myocardial infarction: risk-assessment-based time of hospital discharge vs real life practice. J Clin Med.

[43] Laurencet ME, Girardin F, Rigamonti F, Bevand A, Meyer P, Carballo D, et al (2016) Early Discharge in Low-Risk Patients Hospitalized for Acute Coronary Syndromes: Feasibility, Safety and Reasons for Prolonged Length of Stay. PLoS One. 11(8):e0161493. 10.1371/journal.pone.0161493 10.1371/journal.pone.0161493 PMC4994963 27551861

[44] Ibanez B, James S, Agewall S, Antunes MJ, Bucciarelli-Ducci C et al (2017) ESC guidelines for the management of acute myocardial infarction in patients presenting with ST-segment elevation: The Task Force for the management of acute myocardial infarction in patients presenting with ST-segment elevation of the European Society of Cardiology (ESC). Eur Heart J 2018(39):119–177. 10.1093/eurheartj/ehx393 10.1093/eurheartj/ehx393 28886621

